# Changes in secondary metabolites in the halophytic putative crop species *Crithmum maritimum* L., *Triglochin maritima* L. and *Halimione portulacoides* (L.) Aellen as reaction to mild salinity

**DOI:** 10.1371/journal.pone.0176303

**Published:** 2017-04-25

**Authors:** Christian Boestfleisch, Jutta Papenbrock

**Affiliations:** Institute of Botany, Leibniz University Hannover, Hannover, Germany; Dokuz Eylul Universitesi, TURKEY

## Abstract

It is assumed that salinity enhances the concentration of valuable metabolites in halophytes. The objective was to find a salt concentration and a point in time at which the yield for the valuable metabolites was maximal. Therefore, three different halophyte species were grown under different salinities and harvested over a period from shortly after stress induction up to three weeks. Various reaction patterns were found in the metabolite composition of the analyzed plant material. *Halimione portulacoides* showed a “short term response”, indicated by an increase in all metabolites analyzed after a few hours, whereas *Crithmum maritimum* showed a “long term response” through accumulation of proline starting after days. *Triglochin maritima* did not change in metabolite concentration, but like the other plant species the biomass was reduced by salinity. Generally, a higher production in secondary metabolites by higher salinity was outbalanced by a reduction in biomass production. Concentrations of analyzed antioxidants showed a similar reaction and correlated with each other.

## Introduction

Soil salinity is, besides drought, a major abiotic stress for plants and crops worldwide [1]. Eleven percent of the irrigated area of land which is 34 Mha is affected by salinity and another 60–80 Mha may be affected to some extent due to waterlogging [2]. With an increasing world population, increased harvest and more agricultural land are needed. Otherwise, land which is not suitable for crop production due to salinity may be used by halophytes to produce food and fodder [3,4]. Salinity causes hyperosmotic and hyperionic stress in plants, and osmolytes are produced for protection against hyperosmotic stress [5]. For the protection against oxidative stress, also caused by salinity, antioxidants are produced [6]. The antioxidants can be divided into two groups, antioxidant enzyme systems and non-enzymatic compounds [7]. Part of the non-enzymatic compounds, the secondary plant metabolites include the (poly)phenols consisting of flavonoids and non-flavonoid polyphenols and are present in many vegetables and fruits [8,9]. These antioxidants, among others, are beneficial for human health if consumed [10]. For example, a high intake of (poly)phenols of berries reduces the risk of a cardiovascular disease [11]. Many substances can act as antioxidants whereas the following can be present in plants: vitamin C (ascorbic acid), vitamin E (tocotrienol and tocopherol), carotenoids, (poly)phenols divided in flavonoids and non-flavonoid polyphenols, alkaloids, thiols like glutathione, uric acid and bilirubin [12–14].

As the sum of the abundance and action of many antioxidants, the oxygen radical absorbance capacity (ORAC) can be identified. This determines mainly chain-breaking antioxidants such as (poly)phenols, vitamin C, vitamin E, uric acid and bilirubin [12]. There are two types of ORAC, the hydrophilic ORAC and the lipophilic ORAC. The lipophilic ORAC, determining for example vitamin E and carotenoids, contributes just 5% or often less to the total antioxidant capacity in fruits and vegetables [15,16]. Therefore, often only the hydrophilic ORAC is determined, as in this study.

The three species investigated in this study are already in use for the human diet. *Crithmum maritimum* is consumed by humans since a long time and it is not considered to be toxic if consumed in greater amounts [17,18]. It belongs to the Apiaceae and is a facultative halophyte [19–21]. *Triglochin maritima*, a member of the Juncaginaceae, is edible [22] and an obligate halophyte [23]. *Halimione portulacoides* belongs to the Amaranthaceae and is considered an obligate halophyte [24,25]. It can be consumed raw or cooked [26].

Salinity stress reduces the biomass in vegetable crops, but also increases the secondary metabolite concentration [27,28]. It was previously shown that a manipulation of the antioxidant capacity is possible in certain halophytes [29]. However, the observation time from stress induction to harvest was limited to continuous observation of 24 h (seedlings) or only one harvest after 1, 5 or 8 weeks, for different species, respectively.

The aim of this study was to find the optimal time when the increase of metabolite concentration is still higher than the drawback of reduced growth through salinity. Therefore, plants were harvested after stress induction in the first hours with increasing intervals up to three weeks. So it was possible to determine the exact point of time when antioxidative compounds were produced. It was argued that stress conditions chosen resulting in severe growth reduction are often too harsh. Furthermore experimentally applied stress should not lead to growth reduction of plants [30]. To better understand the mechanisms of salinity, “mild stress conditions” ranging from 0 Practical Salinity Units (PSU is a value derived from conductivity, and is equal to ppt) up to 15 PSU were chosen in this study. Mild stress should not limit the growth of plants significantly compared to severe stress. However, the induced mild stress did not show the anticipated results. There was no point in time at which the concentration of the antioxidants analyzed was higher compared to the gain of biomass. To sum up, the yield of antioxidants was not higher at increased salinity. In *C*. *maritimum* the osmoprotectant proline had a higher concentration and total amount at a salinity of 15 PSU, despite a lower biomass compared to 0 PSU.

## Materials and methods

### Plant material and growth conditions

Seeds of *C*. *maritimum*, *T*. *maritima*, and *H*. *portulacoides* obtained from Rühlemann's (Horstedt, Germany) were germinated on propagation soil (Einheitserde, Einheitserdewerk Hameln-Tündern, Germany), and after 3 to 6 weeks, depending on the season and growth of the species, transplanted to sand (0–2 mm grain size, Hornbach, Hannover, Germany), watered with modified Hoagland solution [31], and finally after 4 to 6 weeks transferred to aerated containers with 13.5 L solution containing the nutrients indicated in S1 Table. After one week, NaCl was added to obtain 0 PSU, 5 PSU, 10 PSU and 15 PSU. For detailed information on culture conditions see Table 1.

**Table 1:** Times for the different nursing steps of the plant species used in the experiments and their origin are shown.

Species	Sawing time to hydroponic culture in weeks	Hydroponic acclimatisation time in weeks	Salinity induction time in h
*C*. *maritimum*	12	2	0, 2, 4, 8, 24, 48, 96, 168, 336, 504
*T*. *maritima*	9	1	0, 2, 4, 8, 24, 48, 96, 168, 336, 504
*H*. *portulacoides*	7	1	0, 2, 8, 24, 96, 336

Mature plants were grown under greenhouse conditions with a minimum temperature of 16.8°C at night-time and maximum of 27.8°C at day-time. The average temperature was 19.3°C. Sodium vapour lamps (SON-T Agro 400, Philips, Amsterdam, Netherlands) served as an additional light source, providing the minimal light flux density (350 μmol m^-2^ s^-1^ for 14 h). Plants were harvested at 0, 2, 4, 8, 24, 48 (2 d), 96 (4 d), 168 (1 week), 336 (2 weeks) and 504 h (3 weeks) after addition of salt, whereas six individuals were harvested at point of time 0 before the addition of salt and 4 individuals per PSU condition were harvested at each other point of time. The complete shoot was taken and individuals were analyzed separately.

### Metabolite extraction

Methods for metabolite extraction were described previously in [29].

Total phenols were measured with Folin-Ciocalteu reagent. Gallic acid was used as standard. The protocol was modified for microtiter plates [32].

Total flavonoids were measured based on a published protocol and modified for mitrotitre plates [33]. After the addition of NaNO_3_, AlCl_3_ and NaOH flavonoids form a coloured complex which was measured at 510 nm. Catechin hydrate was used as standard.

The oxygen radical absorbance capacity (ORAC) assay is based on published protocols [34,35] with modifications [29]. In addition, a mathematical compensation was conducted for the thermal gradient within the plate, which leads to more precise results. The capacity of the sample to prevent fluorescein from being damaged by radicals from 2,2′-azobis(2-amidino-propane) dihydrochloride was measured and compared to the capacity of 6-hydroxy-2, 5,7,8-tetramethylchroman-2-carboxylic acid (Trolox) as standard.

For the determination of AA, dehydroascorbic acid (DHA) and total ascorbic acid (TAA) published protocols [36–38] were modified as described previously [29]. Furthermore small modifications have been made: Metaphosphoric acid (MPA) instead of trichloroacetic acid (TCA) for the extraction and FeCl_3_ of a higher quality were used (sublimed grade, ≥99.9% trace metals basis 701122 Sigma-Aldrich) because the prior use of TCA and a lower quality of FeCl_3_ led to a lower determination rate of AA due to degradation to DHA.

For the determination of proline according to the protocol [39], 50 mg of frozen ground plant material were mixed with 1 ml of 40% ethanol and slowly shaken overnight on an overhead shaker at 4°C. Samples were centrifuged for 5 min at 18,400 *g*. Then 125 μl of supernatant were mixed with 250 μl of reaction mix. The reaction mix consisted of ninhydrin 1% (w/v) in glacial acetic acid 60% (v/v) and ethanol 20% (v/v). The samples were incubated in a water bath at 95°C for 20 min. After cooling to room temperature, 100 μl were transferred into a microtiter plate and read at 520 nm. The results were compared to proline standards ranging from 0.1 to 2 mM.

### Elemental analysis of plant material

Freshly frozen plant material of three individual plants was dried at 80°C for 24 h. Dried plant material was analyzed separately by inductively coupled plasma-optical emission spectroscopy (ICP-OES) based on a published protocol [40]. Concentrations of elements were calculated referring to fresh matter (FM).

### Statistical analysis and evaluation of the data

At each data point four plants (shoots including leaves) were analyzed in triplicate for ORAC, total flavonoids, total phenols, proline and in duplicate for AA. Metabolites were calculated as the mean of four individuals (n = 4). Salinity might have a positive effect on the concentration of some metabolites, but also triggers a lower biomass production. To see whether and when the increase of the concentration outruns the loss of biomass, the total amount (yield) of each metabolite for each point of time was calculated (metabolite concentration multiplied by biomass).

Values were tested for significance with an analysis of variance (ANOVA) using R (version 3.2.2). Significant differences among PSU values were calculated within points of time and among points of time within PSU values. Principal components were calculated in R (version 3.2.2). Pearson correlation was calculated in SigmaPlot (version 12.5).

## Results

The main aim of this study was to find suitable conditions to increase the metabolite concentration while maintaining a high biomass production and an optimal stress induction time period.

### Metabolic changes in *C*. *maritimum*

**Fig 1:**
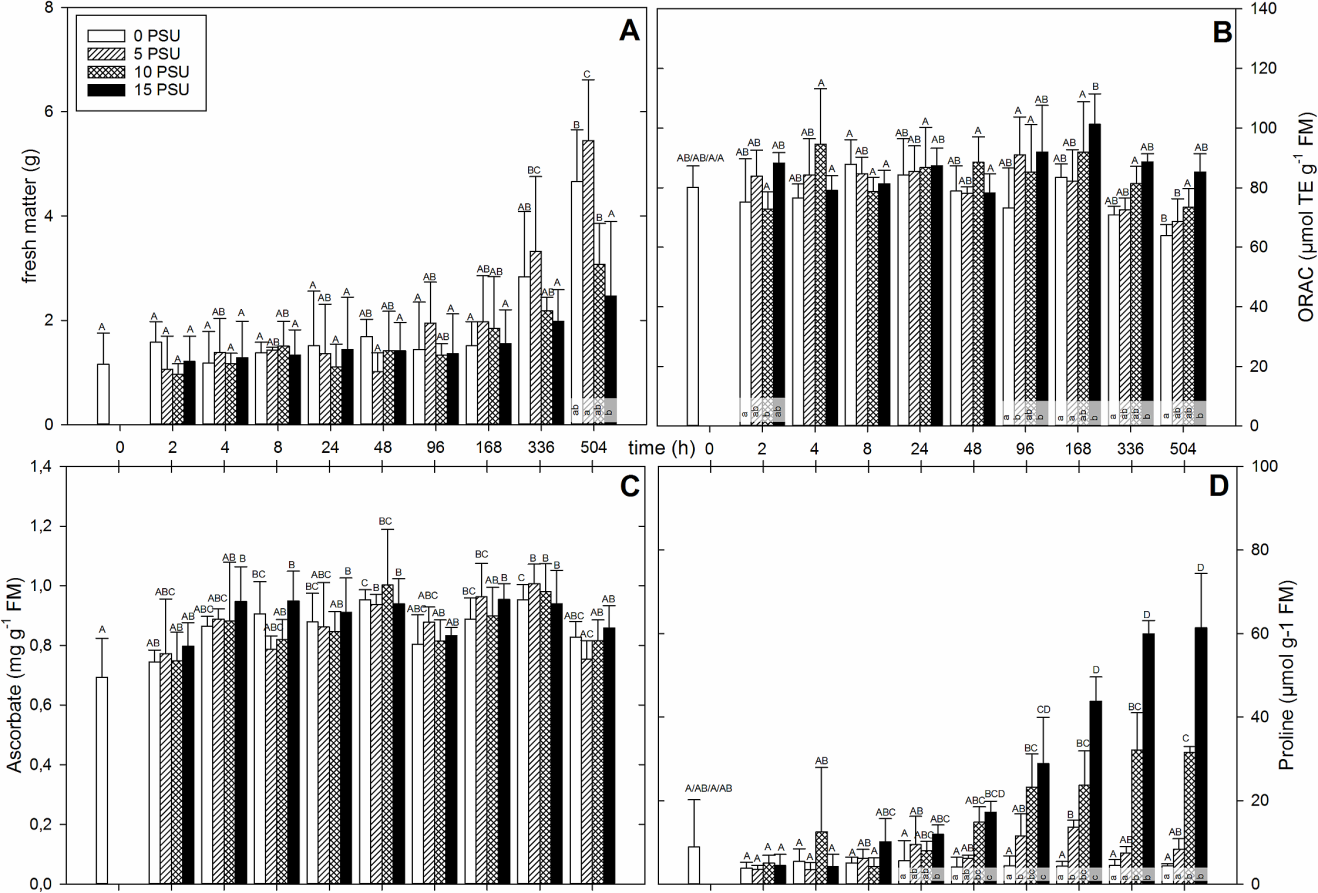
*Crithmum maritimum* plants were set in containers, after an acclimatization time of 2 weeks, 14-week-old plants were exposed to salinity ranging in 5 PSU steps from 0 till 15 PSU. Fresh material (n = 4) was harvested at the indicated time. The mean biomass production (A), ORAC value (B), total ascorbate concentration (C) and proline concentration (D) for each salinity is plotted against the time. For better visibility the time is not true to scale. Different capital letters above the standard deviation indicate significant differences (*p* < 0.05) between points of time among a PSU value. Different lower letters indicate significant differences (*p* < 0.05) within one point of time between different PSU values.

The mean biomass of harvested *C*. *maritimum* plants is shown in Fig 1A. In the beginning the average biomass was 1.16 g per plant. During the first week there were random changes and the fresh matter increased slightly up to around 2 g. The maximum mean biomass per plant was 5.45 g after 504 h at plants grown at 5 PSU. This is significantly different to the mean biomass of plants grown at 15 PSU at the same point of time.

The time pattern for ORAC, total phenols and, as a part of them, total flavonoids of *C*. *maritimum* are significantly correlated with each other (see Table 2 for further information). Therefore, only one metabolite profile is shown and the small differences in between are listed in the text (for the complete data set see Supporting information).

**Table 2:** Analysis of variance of the parameters measured at the different species.

Species	Factor	FM	ORAC	Phenols	Flavonoids	TAA	Proline
*C*. *maritimum*	time	***	***	***	***	***	***
	PSU	** (ns log)	***	***	***	ns	***
	time*PSU	* (ns log)	*	.	ns	ns	***
*T*. *maritima*	time	***	***	***	ns	***	***
	PSU	** (***log)	ns	.	*	ns	***
	time*PSU	* (**log)	ns	*	*	**	***
*H*. *portulacoides*	time	***	***	***	***	***	***
	PSU	ns	***	***	***	***	***
	time*PSU	ns	***	***	**	***	(*log)

The asterisks indicates the significance levels * ≤ 0.05; ** ≤ 0.01; *** ≤ 0.001. n.s. not significant. ANOVA for fresh mass (FM) values was calculated in log.

The ORAC values for *C*. *maritimum* are given in Fig 1B. They started at around 80 μmol TE g^-1^ FM with indistinct changes between time and salinities. There was a general, not significant, increase to the maximum of 101 μmol TE g^-1^ FM in plants grown at 15 PSU in the first week. Afterwards, there was a drop towards values measured in the third week within all plants at all salinities, reaching values of 64 μmol TE g^-1^ FM in plants grown at 0 PSU and 85 μmol TE g^-1^ FM in plants grown at 15 PSU. From 96 h onwards plants grown at 15 PSU had a significantly higher ORAC than plants grown at 0 PSU. There were small differences of total flavonoid concentrations (S1A Fig) in comparison to the ORAC values revealing slightly different trends. There was no increase in the flavonoid concentration in the first 96 h, but the flavonoid concentration at 186 h with plants grown at 10 and 15 PSU stands out more clearly compared to the ORAC values. The total phenol concentration (S1B Fig) had a more distinct slope than the ORAC values up to the maximum at 168 h. Both shared a significant difference at 168 h in between concentrations of plants grown at 0 and 15 PSU.

The amount of DHA was nearly undetectable and the amounts of TAA and AA were nearly the same. Therefore, just TAA values for *C*. *maritimum* are displayed in Fig 1C. The mean TAA concentration increased 2 and 4 h after induction, and increased with fluctuations to a first maximum at 10 PSU after 48 h, followed by a drop down at 96 h. A second rise with a second maximum followed at 336 h after induction. Again there was a decrease in TAA concentration in plants at 504 h. It appeared that at 2 and 4 h after stress induction the plants set to higher salt concentrations had increased TAA concentrations, but this was not significant.

There was no significant change in proline concentration of *C*. *maritimum* in the first hours after salt induction. But from 24 h onwards a significant increase was observed, the proline concentration rose in the plants set to higher salinity (Fig 1D). For plants grown at 15 PSU this trend continued: the proline concentration increased steadily to a plateau at 336 and 504 h. Plants treated with water containing 10 PSU showed a similar significant increase in proline concentration and a plateau at 336 and 504 h but accumulated only about half the amount of plants grown at 15 PSU. Plants set to 5 PSU were only affected slightly and showed only a small increase in proline concentration. The significantly higher maximum was already reached at 168 h after induction, followed by a later decrease in the values. The proline concentration of plants grown at 0 PSU did not change significantly at all.

### Metabolic changes in *T*. *maritima*

**Fig 2:**
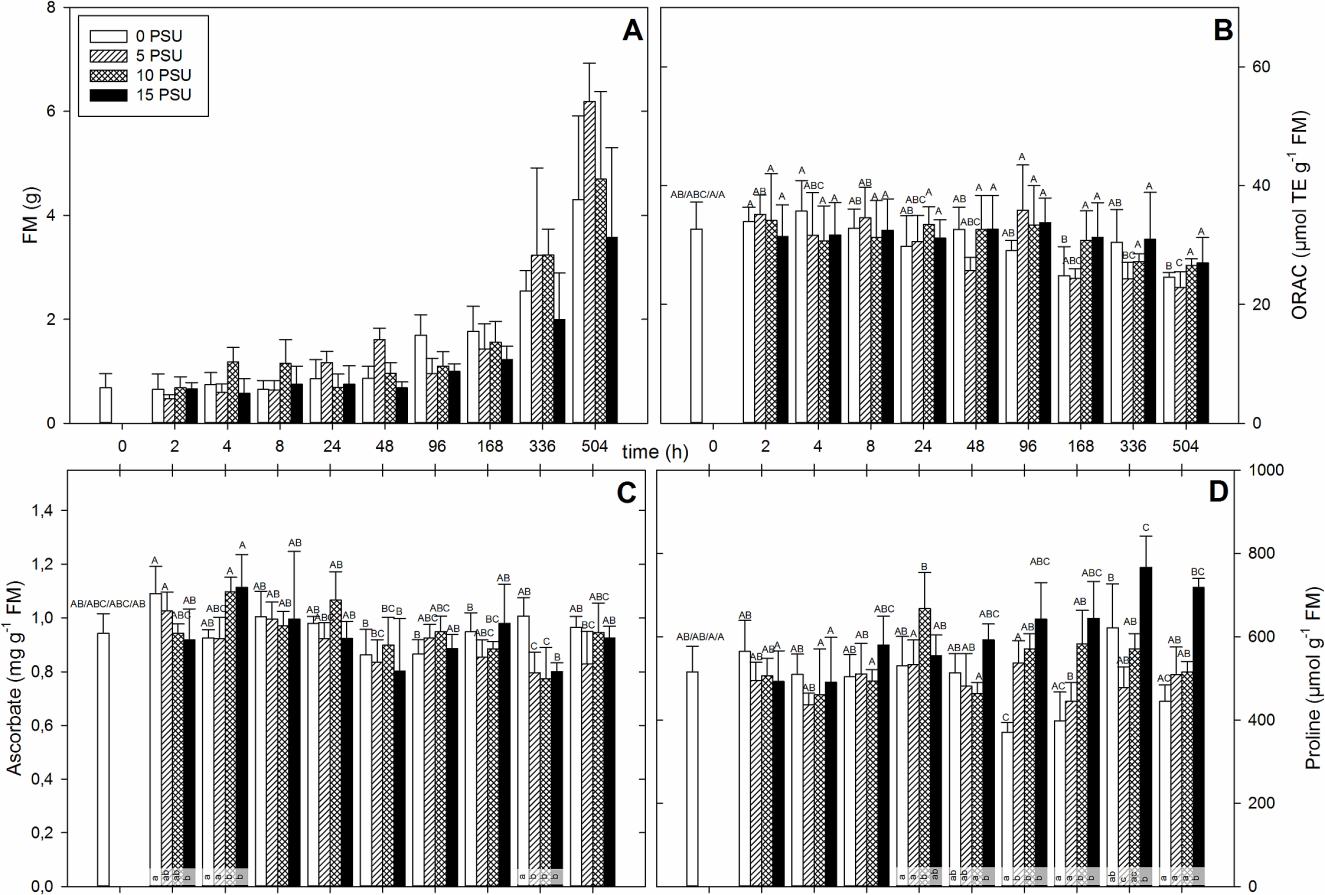
*Triglochin maritima* plants were set in containers, after an acclimatization time of one week, ten week old plants were exposed to salinity ranging in 5 PSU steps from 0 till 15 PSU. Fresh material (n = 4) was harvested at the indicated time. The mean biomass production (A), ORAC value (B), total ascorbate concentration (C) and proline concentration (D) for each salinity is plotted against the time. For better visibility the time is true to scale. Different capital letters above the standard deviation indicate significant differences (*p* < 0.05) between points of time among a PSU value. Different lower letters indicate significant differences (*p* < 0.05) within one point of time between different PSU values.

In Fig 2A the average fresh biomass in g of *T*. *maritima* over time is shown. At the beginning of the experiment there were indistinct changes, but in the later stages, after 336 and 504 h, a correlation between PSU and biomass was observed. The highest mean biomass was harvested after 504 h at plants grown at 5 PSU which was significantly different to plants grown at 15 PSU at the same time and significantly different to the starting value.

ORAC values of *T*. *maritima* are shown in Fig 2B. Starting at 33 μmol TE g^-1^ FM, they decreased over time to 23 up to 27 μmol TE g^-1^ FM in the end. There was some variance in between salinities and points of time, but higher salinities tended to induce higher ORAC values at 168 h and 504 h, however, this was not significant. The temporal patterns of flavonoid and phenol values of *T*. *maritima* appeared similar compared to the temporal pattern of the ORAC values (S2A and S2B Fig). But both flavonoid and phenol decreased in concentration with increasing salinity 2 h after the induction of salinity which was significant for phenol contents. This decrease in metabolite concentration with increasing salinity was only observed at the beginning of the experiment followed by non-significant changes between time and salinities. At 96 and 168 h the plants grown at higher salinities produced higher phenol and flavonoid concentrations. There was a significant difference for phenol concentration at 96 h between plants grown at 0 and 15 PSU. While the range of total phenols remained, with minor exceptions, at the same level over time and only decreased at 336 and 504 h, the span of total flavonoids remained, with minor exceptions, at the same level.

The mean TAA values of *T*. *maritima* are displayed in Fig 2C. The TAA concentration rose 2 h after beginning of the salinity treatment and was significantly higher for plants grown at 0 PSU compared to plants grown at 15 PSU. Just 4 h after induction this constellation changed and plants grown at 10 and 15 PSU had significantly higher values compared to plants grown at 0 and 5 PSU. Afterwards there was a decline without significant changes in the concentration till 48 h for all salinities. The values remained with fluctuations at the same level for the rest of the experiment, except for 336 h where TAA decreased significantly, except for plants grown at 0 PSU.

In *T*. *maritima* the proline mean concentrations did not change significantly within the first 8 h (Fig 2D). At 24 h plants grown at 10 PSU had significantly higher proline concentrations than the other salinities. In the later stages, the proline concentrations of plants grown at 0 and 5 PSU decreased while plants grown at 10 PSU did not change significantly. Plants grown at 15 PSU increased at 336 and 504 h, significantly higher, compared to the starting point and other salinities. This led to a significant increase in the proline concentration of plants grown in higher salinities compared to plants grown in lower salinities at 96 h, 168 h and 504 h and partly at 336 h.

### Metabolic changes *in H*. *portulacoides*

**Fig 3:**
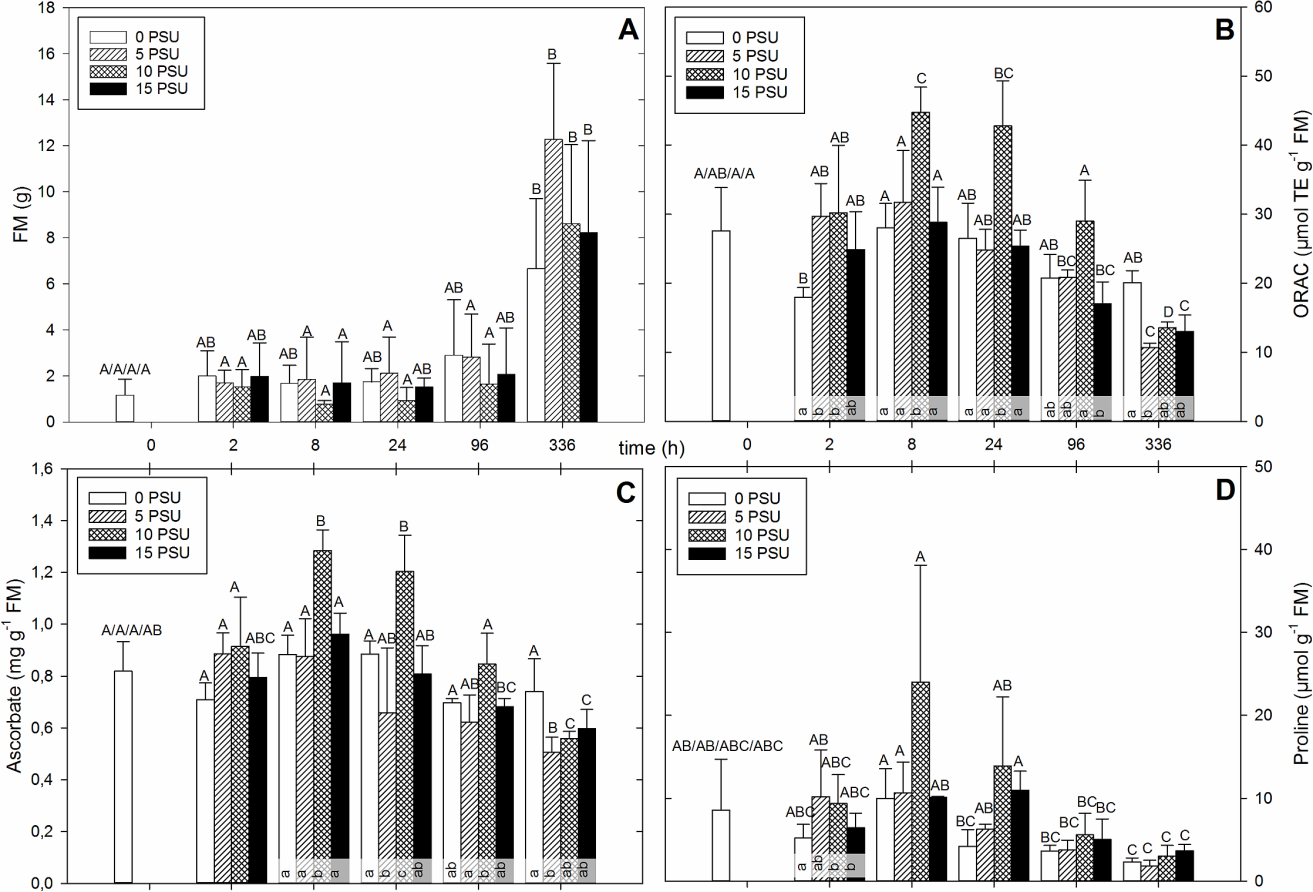
*Halimione portulacoides* plants were set in containers, after an acclimatization time of one week, eight week old plants were exposed to salinity ranging in 5 PSU steps from 0 till 15 PSU. Fresh material (n = 4) was harvested at the indicated time. The mean biomass production (A), ORAC value (B), total ascorbate concentration (C) and proline concentration (D) for each salinity is plotted against the time. For better visibility the time is not true to scale. Different capital letters above the standard deviation indicate significant differences (*p* < 0.05) between points of time among a PSU value. Different lower letters indicate significant differences (*p* < 0.05) within one point of time between different PSU values.

The mean fresh biomass of *H*. *portulacoides* increased slowly during the first four days but increased massively towards the second week (Fig 3A). The highest biomass production was observed in the end of the experiment in plants grown at 5 PSU. There was a significant difference in comparison to the starting value for each salinity, but there was no significant difference in comparison to the last point of time.

The ORAC profile *H*. *portulacoides* is shown in Fig 3B. Eight hours after the induction of salinity stress the ORAC value for plants grown at 10 PSU increased significantly from about 28 up to 45 μmol TE g^-1^ FM. All other ORAC values from plants grown at other salinities at this point of time were not significantly different from the starting point and compared to each other. At 24 h all salinity levels decreased in concentration, but 10 PSU was still significantly higher than the other salinities. This continued at 96 h but was not significant. At 336 h all ORAC values were significant below the starting level except the values for 0 PSU which had a significantly higher concentration compared to the values of the other salinities. The temporal pattern for total flavonoids of *H*. *portulacoides* is given in S3A Fig. The pattern resembles the one of ORAC (Fig 3B) with only little differences. The starting value for the total flavonoid concentrations was higher, so there was no significant difference between that value and the maximum after 8 h at 10 PSU, however, it was still significantly higher compared to the other salinities at the same point of time. At 96 and 336 h there were no significant differences of ORAC values between plants grown at different salinities. The mean total phenol concentration was similar to the ORAC pattern (S3B Fig). After 2 h the phenol concentration of plants grown at 10 PSU was significantly higher than plants grown at 0 PSU but not significantly higher than the starting value. At 8, 24 and 96 h the total phenol concentration was significantly higher in plants grown at 10 PSU compared to the other salinities.

The mean TAA concentration over time for *H*. *portulacoides* is shown in Fig 3C. From the starting value, the concentration rose significantly to a peak at 8 h in plants grown at 10 PSU, while plants at the other salinities did not produce significantly higher concentrations of TAA. At the next point of time of 24 h there was again another significantly higher value in plants grown at 10 PSU, compared to the other salinities. All concentrations declined stepwise at 96 h and 336 h after salinity induction. At 336 h, plants at all salinities except 0 PSU produced values which were significantly lower compared to the starting value.

The temporal pattern of proline for *H*. *portulacoides* is displayed in Fig 3D. After a short time of salt induction (8 h) the amount of proline in salt treated plants (10 PSU) rose higher compared to plants at the other salinities. At 24 h after induction, the proline concentration for salt-exposed plants (10 and 15 PSU) was still higher, but not significant, than the lower stressed and unstressed plants (5 and 0 PSU), but not higher than the starting value. After 96 h the proline concentration decreased to a minimum at 336 h, which was significantly lower, compared to the starting value, for plants grown at 0 and 5 PSU.

### Studies on the dependence of the metabolite concentration from each other

**Fig 4:**
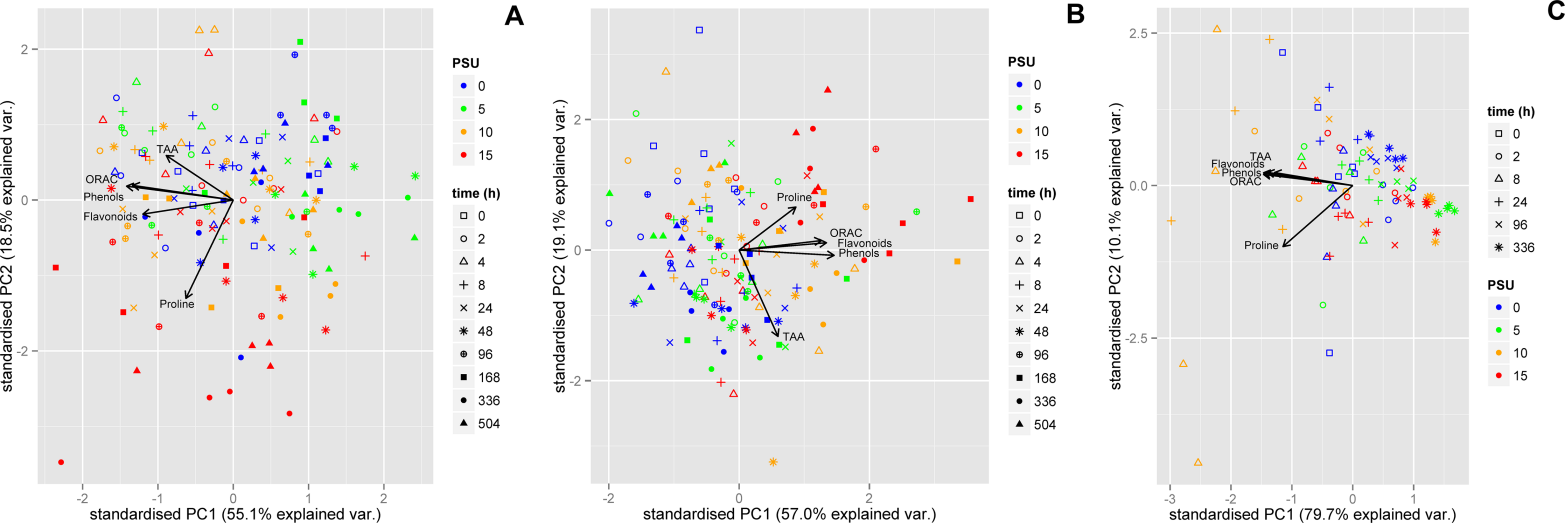
Principal components for individual measurements of ORAC, phenol, flavonoid and proline concentration in *C*. *maritimum* (A), *T*. *maritima* (B) and *H*. *portulacoides*. The different salinities are indicated by different colours and the different points of time by different symbols.

As mentioned earlier some metabolite profiles appeared to be very similar to each other within one species. To visualize the proximity and relation, the Principle component analysis (PCA) was performed and results are displayed in Fig 4A–4C. In Fig 4A the PCA for *C*. *maritimum* is displayed. It is clearly visible, that ORAC, total flavonoid and most of the total phenol values were influenced in the same way in the direction of PC1, whereas proline and TAA values were influenced in different ways, with TAA almost moving along PC2 and proline in the opposite way between PC1 and PC2. A similar result for the PCA of *T*. *maritima* is shown in Fig 4B. The total phenol and the ORAC values were influenced almost in the same way along PC1. TAA values and flavonoids were influenced similarly but shifted in opposite ways more influenced by PC2. Proline values were affected by PC2 and shifted in almost a different direction compared to TAA. Fig 4C shows the PCA of *H*. *portulacoides* and it is obvious that all metabolites, except proline, were affected in a similar way. A clear grouping for time or salinities could not be observed.

To investigate if there is a significant correlation between the different metabolites, the Pearson correlation was calculated (S2 Table). It strengthens the results of the PCA: ORAC, total phenols, and total flavonoids were significantly linearly correlated within *C*. *maritimum*, *T*. *maritima* and *H*. *portulacoides*. There was always a significantly high correlation between ORAC and phenols, whereas in *C*. *maritimum* flavonoids had a higher correlation than phenols towards ORAC. Flavonoids had still a high correlation to ORAC in *T*. *maritima* and *H*. *portulacoides*, in the latter TAA had a higher correlation to ORAC than flavonoids. TAA also had a high correlation to phenols and flavonoids and even proline in *H*. *portulacoides*. The correlation of TAA to the other metabolites was generally lower in *T*. *maritima*, and in *C*. *maritimum* only TAA correlated slightly with phenols. Proline concentration correlated moderately with all other metabolites in *H*. *portulacoides* while in *C*. *maritimum* the correlation was lower and in *T maritima* the correlation was even lower.

The total yield of each metabolite was calculated. The yield of antioxidants was in general highest at the highest biomass production namely at 5 PSU (data not shown). There were some exceptions, for example the gain of proline (Table 3). At 336 h the total gain of proline in *H*. *portulacoides* was highest at the 15 PSU. *Crithmum maritimum* had a significantly higher gain of proline at 48, 96, 168, 336 and 504 h at 15 PSU compared to 0 PSU.

**Table 3:** Amount of proline.

Species	PSU	Time (h)	Proline (μmol g^-1^ FM)	
*H*. *portulacoides*	0	336	14.89	±6.52 a
	5	336	21.12	±4.26 a
	10	336	27.96	±20.92 a
	15	336	30.96	±19.74 a
*C*. *maritimum*	0	48	7.08	±4.48 a
	5	48	6.42	±2.78 a
	10	48	20.32	±9.92 ab
	15	48	24.63	±10.91 b
	0	96	5.68	±2.92 a
	5	96	20.99	±8.82 b
	10	96	30.91	±11.93 b
	15	96	37.04	±16.90 b
	0	168	27.70	±14.37 a
	5	168	6.90	±3.68 b
	10	168	40.33	±18.69 b
	15	168	70.37	±40.00 b
	0	336	14.04	±9.86 a
	5	336	23.78	±7.43 ab
	10	336	69.82	±18.54 bc
	15	336	118.82	±36.68 c
	0	504	20.24	±3.11 a
	5	504	46.37	±19.45 ab
	10	504	96.79	±24.43 b
	15	504	150.23	±87.96 b

The average amount of proline for selected species is calculated and given for points of time, where the yield is higher at high salinity. Different letters in each point of time indicate significant differences (*p* < 0.05).

### Elemental composition

To understand the physiological basis for the differences in the metabolite contents among the plant species, especially of the osmolyte proline, the elemental composition of the halophytes was measured at selected points of time (Table 4). As expected, there was an increase in the Na concentration over time correlating with increasing salinity for each species. Contents of K increased over time in *T*. *maritima* and *H*. *portulacoides* at 0 PSU and decreased at 5, 10 and 15 PSU. In *C*. *maritimum* K contents decreased over time at all conditions. Contents of Ca decreased slowly in all species under salinity. Within the two facultative halophytes the difference of the Ca concentration between the salinity and plants grown at 0 PSU was greater than in the obligate halophyte *C*. *maritimum*. The K/Na ratio decreased in saline conditions for all species, whereas it was almost stable in *C*. *maritimum* and *H*. *portulacoides* at 0 PSU. In *T*. *maritima* the K/Na ratio increased at 0 PSU.

**Table 4:** Elemental composition of the plant material at selected points of time.

Species	Time (h)	PSU	Elemental content in the plant material			
			Na^+^ (mg g^-1^ FM)	K^+^ (mg g^-1^ FM)	Ca^2+^ (mg g^-1^ FM)	K^+^/Na^+^
*C*. *maritimum*	0	0	1.23 ± 0.68	5.65 ± 0.37	4.37 ± 0.22	5.81
	24	0	1.11 ± 0.26	5.85 ± 0.84	4.33 ± 0.30	5.50
		5	1.30 ± 0.14	6.23 ± 2.18	4.69 ± 0.47	4.86
		10	2.55 ± 0.47	4.92 ± 0.43	4.92 ± 0.53	2.00
		15	2.03 ± 0.14	5.2 ± 0.30	5.02 ± 0.21	2.57
	96	0	0.89 ± 0.24	4.71 ± 0.52	3.64 ± 0.49	5.57
		5	1.92 ± 0.38	4.8 ± 0.03	3.77 ± 0.16	2.54
		10	2.86 ± 0.86	4.83 ± 1.05	4.12 ± 0.71	1.86
		15	3.54 ± 1.06	4.43 ± 0.87	4.09 ± 0.24	1.40
	336	0	0.88 ± 0.15	4.39 ± 0.56	3.26 ± 0.15	5.12
		5	2.91 ± 0.44	4.27 ± 0.83	2.91 ± 0.30	1.46
		10	3.46 ± 0.40	3.77 ± 0.73	3.17 ± 0.44	1.11
		15	4.97 ± 0.29	4.19 ± 1.38	3.72 ± 0.32	0.84
*T*. *maritima*	0	0	3.55 ± 0.54	4.43 ± 1.03	2.16 ± 0.29	1.24
	96	0	2.59 ± 0.33	6.91 ± 2.63	2.14 ± 0.17	2.67
		5	6.8 ± 1.69	5.74 ± 1.99	2.1 ± 0.36	0.87
		10	6.96 ± 0.73	5.2 ± 1.34	2.15 ± 0.17	0.76
		15	6.92 ± 0.87	5.9 ± 1.48	2.75 ± 0.68	0.84
	336	0	2.53 ± 0.21	9.63 ± 1.33	3.45 ± 0.52	3.84
		5	6.89 ± 0.26	4.21 ± 1.54	1.77 ± 0.23	0.62
		10	9.05 ± 0.42	3.91 ± 0.4	1.43 ± 0.08	0.43
		15	10.78 ± 0.85	4 ± 0.85	1.4 ± 0.05	0.37
*H*. *portulacoides*	0	0	4.9 ± 0.76	6.67 ± 1.77	2.24 ± 0.27	1.42
	96	0	5.25 ± 2.23	7.43 ± 4.09	2.35 ± 0.66	1.76
		5	8.66 ± 0.74	5.27 ± 2.25	1.69 ± 0.14	0.62
		10	7.96 ± 0.69	5.2 ± 1.79	1.73 ± 0.51	0.64
		15	9.65 ± 0.63	6.45 ± 0.76	1.53 ± 0.27	0.67
	336	0	5.13 ± 1.6	7.74 ± 1.22	1.89 ± 0.22	1.58
		5	8.72 ± 0.81	6.37 ± 0.45	0.85 ± 0.08	0.74
		10	9.76 ± 0.54	6.36 ± 2.01	0.74 ± 0.09	0.65
		15	10.94 ± 0.66	4.07 ± 0.59	0.72 ± 0.15	0.37

Mean (± S.D.) are given (n = 3). For a better comparison the Na^+^ in the hydroponic solution is calculated: 0, 5, 10 and 15 PSU equals 0, 1.97, 3.93 and 5.90 mg g^-1^ FM.

## Discussion

Many studies agree that there is a health-promoting effect from antioxidants of various plant sources [8,9,41]. It was also shown that salinity stress causes an increase in the concentration of antioxidants [6,42]. Thus, mild salt-stress might produce healthier vegetables containing high amounts of antioxidants.

*Crithmum maritimum* showed a significant increase in ORAC, total phenols and flavonoid concentration at 168 h after salinity induction comparing the contents at 0 and 15 PSU. This was also the point of time when the plants accumulated the highest concentrations of these antioxidants. Therefore, this would be the best point of time to harvest. This consideration leaves out the concentration of proline and TAA, because proline is of low economic value and the TAA values were not significantly different between salinities. *Triglochin maritima* showed an increased antioxidant production with increased salinity, but at different points of time as the maxima in antioxidant concentrations vary. The maximum ORAC value was measured after 96 h in plants gown at 5 PSU and the maximum flavonoid concentration at 336 h in plants grown at 15 PSU, however, there was no significant difference in comparison to plants grown at 0 PSU. The only significant maximum was determined at 96 h in the phenol concentration in plants grown at 15 PSU. TAA concentrations accumulated to a significant maximum at 4 h in plants grown at 15 PSU; therefore, there was no optimal point of time for a harvest. In the case of *H*. *portulacoides* it is very conclusive: the best point of time for a harvest would be 8 h after salinity induction for plants grown at 10 PSU. At this point of time there were significant differences in ORAC, total phenol, total flavonoid and TAA concentrations.

However, taking the biomass for the total antioxidant yield into consideration, the best point of time for a harvest shifts. Within one week, biomass production was not severely inhibited by salinity, however, there was a trend to a decreased biomass production in plants grown at 10 and 15 PSU. This trend continues at 336 h after salinity induction and an actual increase of biomass production of plants grown at 5 PSU became obvious. At 504 h after salinity induction there was a significant difference in the gain of biomass of *C*. *maritimum* and *T*. *maritima* between the maximum, in plants grown at 5 PSU, and the lowest gain of biomass, in plants grown at 15 PSU. A higher gain of biomass, yields a higher amount of secondary metabolites. A high antioxidant activity and a high biomass production under the same condition did not coincide, furthermore, the lower biomass production compensated the increased antioxidant concentration.

Drought stress causes a complex pattern of reactions in plants regulated by the activity of many genes [43]. Often transcriptome dynamics are in consistence enzymatic and metabolic changes [44]. The modified enzyme patterns result in an increase of antioxidants influencing, for example, stomata closing to prevent further loss of water but also preventing further uptake of CO_2_ [28]. Therefore, reduction equivalents like NADPH+H^+^ are increased, as they are not consumed for CO_2_-fixation in the Calvin cycle. Thus, metabolic processes are directed to produce highly reduced secondary metabolites, such as isoprenoids, (poly)phenols or alkaloids. But the decrease of biomass under drought stress revises the yield in many cases [28]. The mechanisms of stomatal closure due to water deficiency are comparable under salinity and drought stress. However, the closed stomata preventing further CO_2_-fixation, affect the growth of the plant. To work around the problem of biomass reduction, mild salinity conditions were chosen. But mild salinity obviously does not increase the yield of secondary plant products in halophytes to a high extent to overcome the reduction in biomass.

The yield of proline, which acts as an osmolyte, was different compared to the antioxidants. There was a significant increase in proline concentration with increasing salinity for all three species. This increase of the total yield was sufficient to compensate for the loss of biomass production, at least for *C*. *maritimum* and *H*. *portulacoides*. In *H*. *portulacoides* the total proline yield was the highest after 336 h at 15 PSU, however, this was not significant. In *C*. *maritimum* the proline yield was significantly higher at 15 PSU from 48 h onwards (Table 3). The search for halophytes containing valuable osmolytes increased by salinity with a high probability might be a promising future approach. There are already some pharmaceutical and medicinal applications for osmolytes, for example protection against carcinogenic oxidative stress, helping in vaccine stabilisation and treating the dry eye syndrome, for the complete list see the review [45].

A correlation between salinity stress and an increase in proline in halophyte species and therefore the use as a stress marker was postulated previously [46–48]. Also in glycophytes a proline up-regulation was shown, but under drought stress [49]. There are several other osmolytes present in halophytes [50], which means that both *H*. *portulacoides* and *T*. *maritima* might use other types of osmolytes for protection. In *H*. *portulacoides* high concentrations of glycine betaine were found in plants harvested in a salt marsh [51], as well as methylated onium compounds and sugars [45]. *Triglochin maritima* does not seem to significantly increase any specific osmolyte concentration [52]. Many osmolytes like glycine betaine and sugars were found in a moderate concentration compared to other monocotyledonous salt marsh species. Proline, pipecolate, fructose, maltose, and sucrose were also found in *T*. *maritima* [45]. The ten times higher proline concentration, in comparison to the other plant species investigated, which was already present at non-saline conditions, seems to be sufficient for osmotic balance. Furthermore, it was shown that the synthesis of osmolytes might be constitutive in some species as the concentration of osmolytes does not increase with increasing salinity in all cases [45].

On average, the nutritional value of the halophytes is high compared to other raw vegetables. The ORAC value of *C*. *maritimum* was around 80 μmol TE g^-1^ FM which is comparable to red pepper [12]. The ORAC values of *T*. *maritima* and *H*. *portulacoides* were around 20–40 μmol TE g^-1^ FM. These values are comparable to raw asparagus or raw broccoli [15]. The total phenol concentration of *C*. *maritimum* is around 3 mg GAE g^-1^ FM, comparable to red [52] or green pepper and broccoli [15]. *Triglochin maritima* and *H*. *portulacoides* have total phenol values which are comparable with asparagus, carrots, yellow onions [15] or spinach and broccoli [53]. It is important to mention that the values of the reference sources vary, but this is most likely due to the collection time, season, variety and other reasons.

It is notable that the Na^+^ concentration of *C*. *maritimum* under salinity was always lower compared to the concentration of the surrounding culturing solution. On the one hand, this might indicate that the plant acts as an excluder to keep Na^+^ out. On the other hand, this might result in an osmotic pressure, and to prevent this, the proline concentration is enhanced. The Na^+^ concentrations of *T*. *maritima* and *H*. *portulacoides* are higher than in the surrounding media. *Triglochin maritima* is described as leaf-succulent halophyte acting as an excretive halophyte with glandular cells [54,55]. *Halimione portulacoides* has epidermal bladders to remove the salt, but it also accumulates a high concentration in the leaves to maintain an osmotic balance [56], as it was also confirmed by our results (Table 3). Whether the salt was transported to glandular cells or epidermal bladders cannot be discriminated as the whole plant material was analyzed.

The similar trends with respect to time in the biosynthesis of compounds with high ORAC, total phenols and total flavonoids have multiple reasons. As mentioned earlier, flavonoids are one of the two groups of (poly)phenols [8,9]. When the phenols were measured, the flavonoids were automatically measured as well, contributing half or two thirds to the amount of phenols, leading to the similar courses of these metabolites. As the ORAC value represents the sum of the most abundant antioxidants available in the plant there is a similarity towards phenols and therefore also flavonoids. This can be seen in *C*. *maritimum* having high flavonoid values in comparison to phenols, therefore, both values have a high correlation. The high phenol concentration contributes more to the ORAC value than the TAA concentration leading to a high correlation between ORAC and phenols (and therefore flavonoids), but a low correlation with ORAC and TAA. *Halimione portulacoides* has a relatively high TAA concentration in relation to phenols. Therefore, TAA is contributing more to the ORAC resulting in a higher correlation between them. A positive correlation between phenolics and antioxidant activity, degree of correlation varying upon analyzed material, was reported before [57,58]. Furthermore a correlation between ORAC, total phenolic and TAA content was found in soybean leaves [35]. We could also observe a correlation between all measured metabolites, partly weaker and partly stronger, depending on the plant species. Another study postulated that the correlation between AA and ORAC is high, when the AA concentration is high [59]. They analyzed guava fruits with an even or higher AA concentration than (poly)phenol concentration. In analyzed *Vaccinium* species with a high phenol concentration and an around 50 times lower AA concentration the AA only contributed 5% or less to the ORAC [60].

The size of the plants has to be taken into account. Some smaller plants showed higher concentration in secondary compounds and vice versa. This natural variety influences the ORAC and phenol values in *C*. *maritimum* and *T*. *maritima*, furthermore the TAA in the latter and all values in *H*. *portulacoides*. This leads to a fluctuation in all metabolite patterns also in the control plants grown at 0 PSU. A correlation between the secondary metabolite camptothecin and tissue age was found, and a correlation between the phlorotannin content and size of kelp, summarizing that secondary metabolites are synthesized at the cost of growth [61,62].

## Conclusion

In our study mild stress promoted content of secondary metabolites partly after a few hours, partly after several days. But in all cases the biomass reduction in plants grown in mild stress exceeded the increase in metabolite concentration in mildly stressed plants resulting in an equal or even lower yield of metabolites per cultivated area. Therefore, the total amount of secondary metabolites per harvest was lower in the stressed plants, except for proline. There might be other individual metabolites affected in the same way. So far, total (poly)phenol concentrations were taken into consideration, but analyzing individual valuable phenolic compounds is a promising future approach because single components might react to salinity. Salinity-affected soils occur all over the world. These lands are not suitable for conventional agriculture, but for saline agriculture. Species that can be grown in these environments are halophytes. It was shown that it is possible to manipulate their antioxidant concentration and enhance their value. As salt affected areas are a general problem already, halophytes can be grown on saline areas where glycophytic crops do not survive. Alternatively, halophytes could be cultivated in hydroponic greenhouse conditions by using brackish water to enhance their nutritional value.

## Supporting information

S1 Fig *Crithmum maritimum* plants were set in containers, after an acclimatization time of 2 weeks, 14-week-old plants were exposed to salinity ranging in 5 PSU steps from 0 till 15 PSU. Fresh material was harvested at the indicated time. The mean flavonoid (A) and phenol concentration (B) out of four plants for each salinity is plotted against the time. For better visibility the time is not scaled natural. Different capital letters above the standard deviation indicate significant differences (*p* < 0.05) between points of time among a PSU value. Different lower letters indicate significant differences (*p* < 0.05) within one point of time between different PSU values.
(TIF)

S2 Fig *Triglochin maritima* plants were set in containers, after an acclimatization time of 2 weeks, 14-week-old plants were exposed to salinity ranging in 5 PSU steps from 0 till 15 PSU. Fresh material was harvested at the indicated time. The mean flavonoid (A) and phenol concentration (B) out of four plants for each salinity is plotted against the time. For better visibility the time is not scaled natural. Different capital letters above the standard deviation indicate significant differences (*p* < 0.05) between points of time among a PSU value. Different lower letters indicate significant differences (*p* < 0.05) within one point of time between different PSU values.
(TIF)

S3 Fig *Halimione portulacoides* plants were set in containers, after an acclimatization time of 2 weeks, 14-week-old plants were exposed to salinity ranging in 5 PSU steps from 0 till 15 PSU. Fresh material was harvested at the indicated time. The mean flavonoid (A) and phenol concentration (B) out of four plants for each salinity is plotted against the time. For better visibility the time is not scaled natural. Different capital letters above the standard deviation indicate significant differences (*p* < 0.05) between points of time among a PSU value. Different lower letters indicate significant differences within one point of time between different PSU values.
(TIF)

S1 Table Composition of the nutrient solution. (DOCX)

S2 Table Pearson correlation of four assays for each species. The number indicates the correlation coefficient, the asterisk indicates the significance levels * ≤ 0.05; ** ≤ 0.01; *** ≤ 0.001. n.s. not significant; TAA, total ascorbic acid.
(DOCX)

## Abbreviations

AA: ascorbic acid
CE: catechin equivalent
DHA: dehydroascorbic acid
FeCl_3_: ferric chloride
FM: fresh matter
GAE: gallic acid equivalent
ICP-OES: inductively coupled plasma atomic emission spectroscopy
MPA: metaphosphoric acid
ORAC: oxygen radical absorbance capacity
PC: principal component
PSU: practical salinity units
TAA: total ascorbic acid
TCA: trichloroacetic acid
TE: trolox equivalent
