# Supplementary figures and images for: Changes in secondary metabolites in the halophytic putative crop species Crithmum maritimum L., Triglochin maritima L. and Halimione portulacoides (L.) Aellen as reaction to mild salinity
Source: PLoS One. 2017 Apr 25;12(4):e0176303. doi: 10.1371/journal.pone.0176303 (PMC5404854; doi:10.1371/journal.pone.0176303)

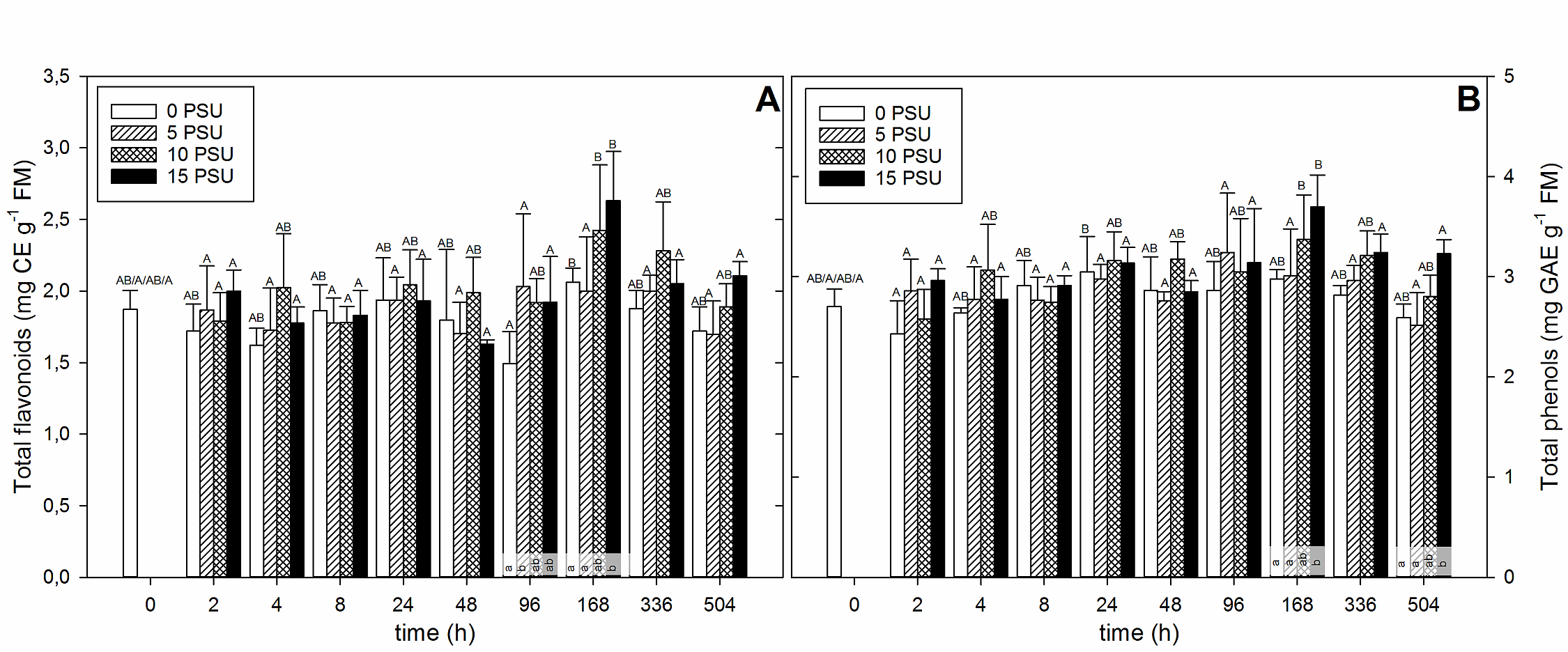

Supplement: S1 Fig — Fresh material was harvested at the indicated time. The mean flavonoid (A) and phenol concentration (B) out of four plants for each salinity is plotted against the time. For better visibility the time is not scaled natural. Different capital letters above the standard deviation indicate significant differences (p < 0.05) between points of time among a PSU value. Different lower letters indicate significant differences (p < 0.05) within one point of time between different PSU values. (TIF) [file pone.0176303.s001.tif]

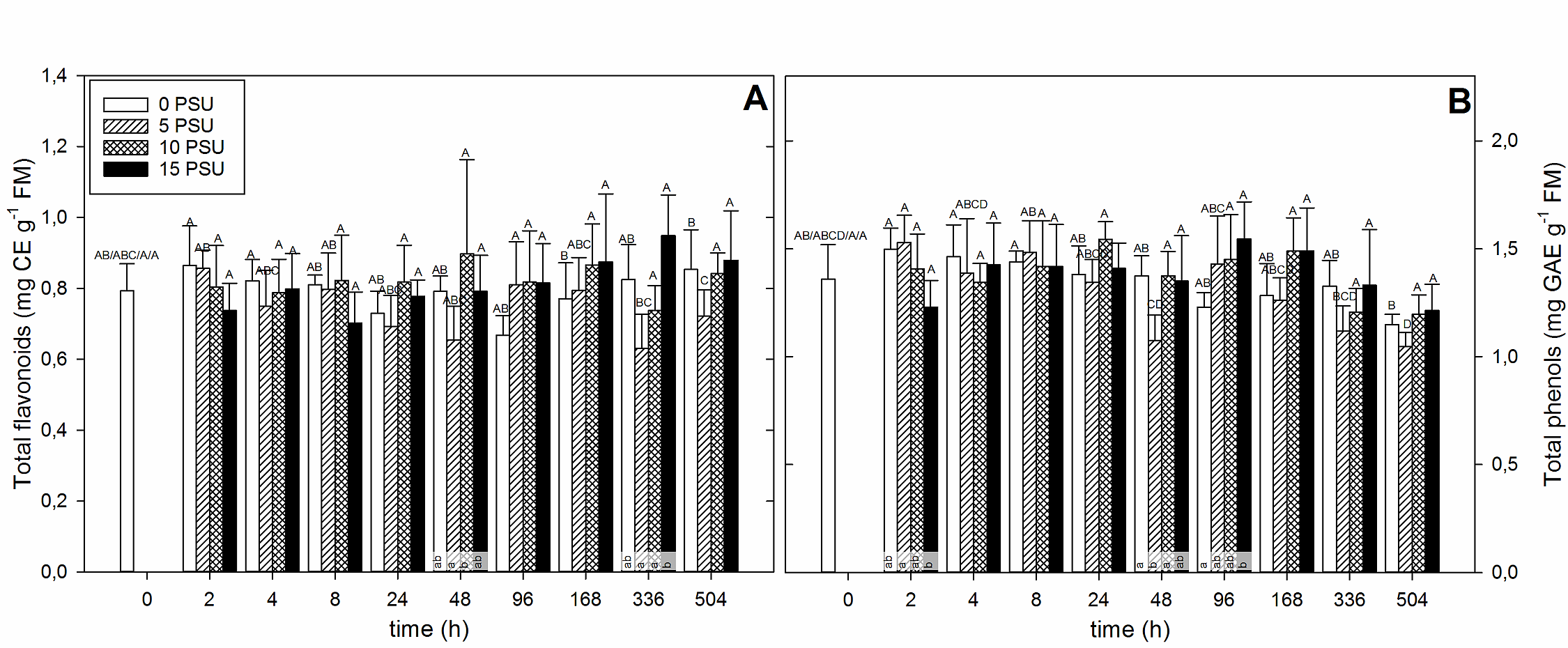

Supplement: S2 Fig — Fresh material was harvested at the indicated time. The mean flavonoid (A) and phenol concentration (B) out of four plants for each salinity is plotted against the time. For better visibility the time is not scaled natural. Different capital letters above the standard deviation indicate significant differences (p < 0.05) between points of time among a PSU value. Different lower letters indicate significant differences (p < 0.05) within one point of time between different PSU values. (TIF) [file pone.0176303.s002.tif]

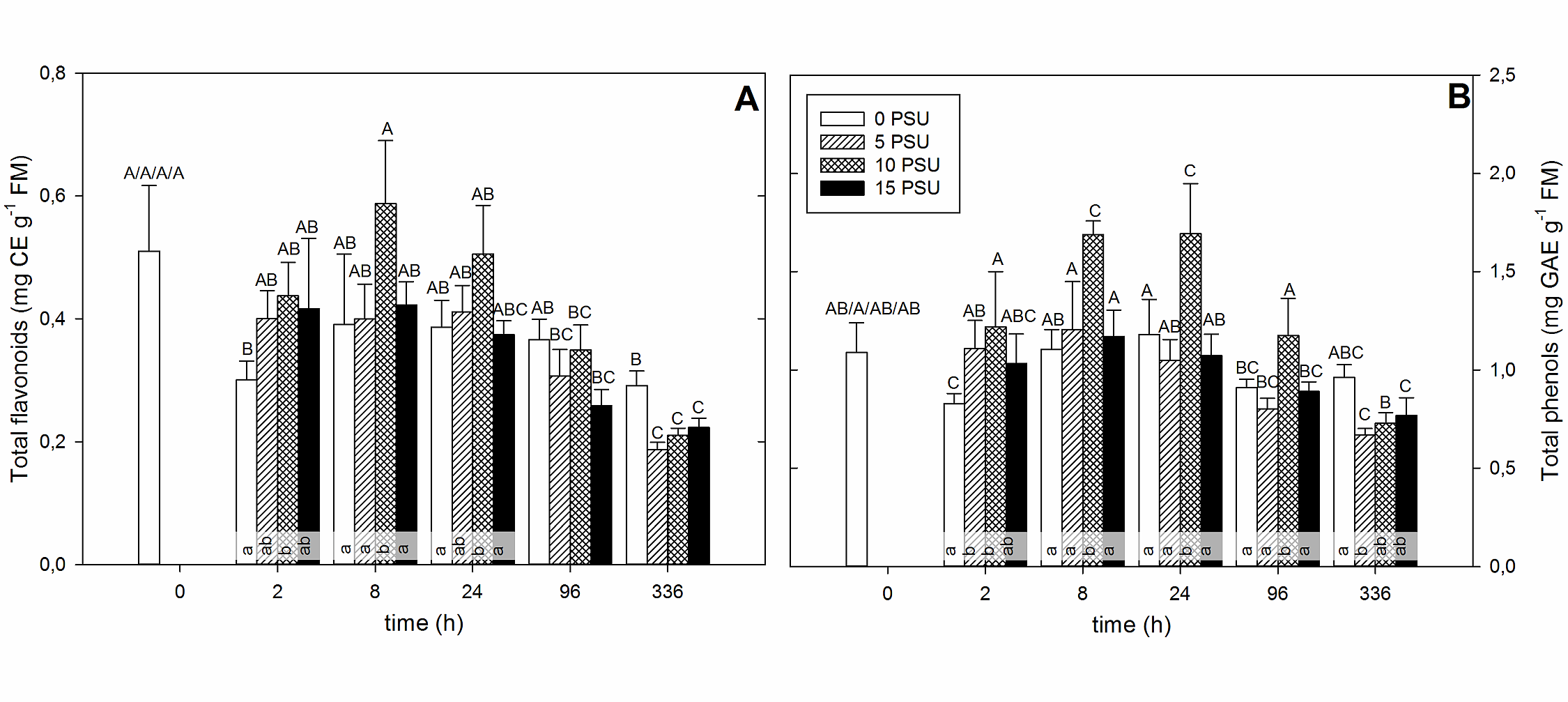

Supplement: S3 Fig — Fresh material was harvested at the indicated time. The mean flavonoid (A) and phenol concentration (B) out of four plants for each salinity is plotted against the time. For better visibility the time is not scaled natural. Different capital letters above the standard deviation indicate significant differences (p < 0.05) between points of time among a PSU value. Different lower letters indicate significant differences within one point of time between different PSU values. (TIF) [file pone.0176303.s003.tif]
