# Supplementary material for: Changes in secondary metabolites in the halophytic putative crop species Crithmum maritimum L., Triglochin maritima L. and Halimione portulacoides (L.) Aellen as reaction to mild salinity
Source: PLoS One. 2017 Apr 25;12(4):e0176303. doi: 10.1371/journal.pone.0176303 (PMC5404854; doi:10.1371/journal.pone.0176303)
Supplement: S1 Table — (DOCX) [file pone.0176303.s004.docx]

**S1 Table.** Composition of the nutrient solution.

| **Chemical compound** | **mg*l^-1^** | **µmol*l^-1^** |
| --- | --- | --- |
| KNO_3_ | 606.60 | 6000 |
| Ca(NO_3_)_2_ x 4H_2_O | 944.64 | 4000 |
| NH_4_H_2_PO_4_ | 230.16 | 2000 |
| MgSO_4_ x 7H_2_O | 246.48 | 1000 |
| KCl | 3.73 | 50 |
| H_3_BO_3_ | 1.55 | 25 |
| MnSO_4_ x H_2_O | 0.34 | 2.0 |
| ZnSO_4_ x 7H_2_O | 0.58 | 2.0 |
| CuSO_4_ x 5H_2_O | 0.12 | 0.5 |
| MoNa_2_O_4_ x 2H_2_O | 0.12 | 0.5 |
| C_10_H_16_FeN_2_NaO_8_ | 0.60 | 10 |
