# Supplementary material for: Changes in secondary metabolites in the halophytic putative crop species Crithmum maritimum L., Triglochin maritima L. and Halimione portulacoides (L.) Aellen as reaction to mild salinity
Source: PLoS One. 2017 Apr 25;12(4):e0176303. doi: 10.1371/journal.pone.0176303 (PMC5404854; doi:10.1371/journal.pone.0176303)
Supplement: S2 Table — The number indicates the correlation coefficient, the asterisk indicates the significance levels * ≤ 0.05; ** ≤ 0.01; *** ≤ 0.001. n.s. not significant; TAA, total ascorbic acid. (DOCX) [file pone.0176303.s005.docx]

**S2 Table.** Pearson correlation of four assays for each species. The number indicates the correlation coefficient, the asterisk indicates the significance levels * ≤ 0.05; ** ≤ 0.01; *** ≤ 0.001. n.s. not significant; TAA, total ascorbic acid.

| **Species** | **Metabolites** | **Flavonoids** | | **Phenols** | | **TAA** | | **Proline** | |
| --- | --- | --- | --- | --- | --- | --- | --- | --- | --- |
| *C. maritimum* | ORAC | 0.632 | *** | 0.781 | *** | 0.16 | n.s. | 0.297 | *** |
|  | flavonoids |  |  | 0.818 | *** | 0.227 | n.s. | 0.407 | *** |
|  | phenols |  |  |  |  | 0.378 | *** | 0.449 | *** |
|  | TAA |  |  |  |  |  |  | 0.062 | n.s |
| *T. maritima* | ORAC | 0.577 | *** | 0.86 | *** | 0.344 | *** | 0.206 | * |
|  | flavonoids |  |  | 0.626 | *** | 0.295 | *** | 0.296 | *** |
|  | phenols |  |  |  |  | 0.475 | *** | 0.262 | ** |
|  | TAA |  |  |  |  |  |  | 0.113 | n.s |
| *H. portulacoides* | ORAC | 0.775 | *** | 0.966 | *** | 0.874 | *** | 0.643 | *** |
|  | flavonoids |  |  | 0.755 | *** | 0.694 | *** | 0.552 | *** |
|  | phenols |  |  |  |  | 0.902 | *** | 0.632 | *** |
|  | TAA |  |  |  |  |  |  | 0.598 | *** |
